# Transgenic Fluorescent *Plasmodium cynomolgi* Liver Stages Enable Live Imaging and Purification of Malaria Hypnozoite-Forms

**DOI:** 10.1371/journal.pone.0054888

**Published:** 2013-01-24

**Authors:** Annemarie Voorberg-van der Wel, Anne-Marie Zeeman, Sandra M. van Amsterdam, Alexander van den Berg, Els J. Klooster, Shiroh Iwanaga, Chris J. Janse, Geert-Jan van Gemert, Robert Sauerwein, Niels Beenhakker, Gerrit Koopman, Alan W. Thomas, Clemens H. M. Kocken

**Affiliations:** 1 Biomedical Primate Research Centre, Department of Parasitology, Rijswijk, The Netherlands; 2 Mie University, School of Medicine, Tsu, Japan; 3 Leiden Malaria Research Group, Department of Parasitology, Leiden University Medical, Leiden, The Netherlands; 4 Department of Medical Microbiology, University Medical Centre, Nijmegen, The Netherlands; 5 Biomedical Primate Research Centre, Department of Virology, Rijswijk, The Netherlands; Université Pierre et Marie Curie, France

## Abstract

A major challenge for strategies to combat the human malaria parasite *Plasmodium vivax* is the presence of hypnozoites in the liver. These dormant forms can cause renewed clinical disease after reactivation through unknown mechanisms. The closely related non-human primate malaria *P. cynomolgi* is a frequently used model for studying hypnozoite-induced relapses. Here we report the generation of the first transgenic *P. cynomolgi* parasites that stably express fluorescent markers in liver stages by transfection with novel DNA-constructs containing a *P. cynomolgi* centromere. Analysis of fluorescent liver stages in culture identified, in addition to developing liver-schizonts, uninucleate persisting parasites that were atovaquone resistant but primaquine sensitive, features associated with hypnozoites. We demonstrate that these hypnozoite-forms could be isolated by fluorescence-activated cell sorting. The fluorescently-tagged parasites in combination with FACS-purification open new avenues for a wide range of studies for analysing hypnozoite biology and reactivation.

## Introduction

*Plasmodium vivax* is the most widely distributed cause of human malaria having an enormous socio-economical impact with an estimated 132 to 391 million clinical cases per year [1]. There is an increased awareness of the severity of the disease that *P. vivax* can cause [2], [3], yet radical cure of *P. vivax* infections is hampered by the existence of hypnozoites, which are dormant forms present in the liver that can cause blood stage infections upon reactivation [4]. Hypnozoites are insensitive to most anti-malarial drugs that kill developing blood- and liver stages [5]. Primaquine is currently the only available drug that kills the dormant hypnozoites, but its severe side effects in glucose-6-phosphate dehydrogenase (G6PD)-deficient people prevent the widespread use of the drug [6]. The presence of hypnozoites and their drug-insensitivity form a major hurdle for elimination programmes and it is generally agreed that the mission to eradicate malaria initiated by Bill and Melinda Gates [7] can only be successful if effective means exist to remove this hidden reservoir of hypnozoites from the population [5], [8].

Despite the importance of hypnozoites for initiating relapse-infections hardly anything is known about their biology and the mechanisms underlying dormancy and reactivation of these forms. This is mainly due to the absence of robust *in vitro* culture systems not only for liver stages (including hypnozoites), but also for any other *P. vivax* life cycle stage [9]. Recently a small-scale liver culture system for *P. vivax*, relying on cryopreserved sporozoites has been described [10], and small forms expressing CSP were seen at day 9. However, more work is needed to demonstrate that these forms are hypnozoites.

Access to *P. vivax* sporozoites and *P. vivax* relapse research *in vivo* in animal models is severely hampered by its host range that is restricted to some New World monkey species and chimpanzees [2], [8]. Consequently, much of the knowledge on the biology of *vivax*-type parasites is derived from studies using a closely related non-human primate malaria parasite *Plasmodium cynomolgi* [11]. This parasite also forms hypnozoites and has been the gold-standard *in vivo* model for studying relapse-infections that result from reactivation of hypnozoites [2]. Recently, technologies have been developed for the in vitro cultivation of the liver stages of *P. cynomolgi*. Similar to what was observed in *P. vivax* [10], this included forms that resemble hypnozoites [12].

*In vitro* cultures of *Plasmodium* liver stages in which hypnozoite-forms are produced are exciting developments offering new possibilities to investigate the biology of hypnozoites and, importantly, for screening drugs that target these forms. However, these analyses need to be robust and amenable to high throughput methodologies, and currently this can only realistically be achieved through genetic modification of the *P. cynomolgi* genome, whereby it is possible to create transgenic reporter parasites and gene-deletion mutants as has been shown for other *Plasmodium* parasites [13]–[19]. Thus far transfection technology for *P. cynomolgi* is not well developed [20], [21] and transgenic parasites expressing fluorescent markers for analysis of liver stages are not available. Recently the use of a *Plasmodium* artificial chromosome (PAC) as transfection tool has been reported for the rodent malaria *P. berghei* [22]. In this study it was shown that transfection with DNA constructs containing a centromeric sequence results in stable maintenance and segregation of both circular and linear DNA constructs throughout the complete life cycle including mosquito transmission and liver stage development. We have adopted this technology to generate for the first time *P. cynomolgi* mutant parasites that stably express fluorescent reporters in liver stages. These reporter parasites were generated by transfection using a novel DNA-construct that contains a *P. cynomolgi* centromeric sequence and two reporter proteins, mCherry and GFP under the control of two different promoters. Analysis of fluorescent liver stages of these reporter parasites identified developing liver-schizonts and fluorescent, uninucleate persisting forms that showed all characteristics of hypnozoite-forms. Importantly, we were able to sort these hypnozoites-forms by fluorescence-activated cell sorting (FACS) based on their GFP-fluorescence intensity. These reporter lines and technologies to isolate hypnozoite-forms provide new tools not only to analyse hypnozoite biology and reactivation but also for larger scale screening of drugs that target hypnozoites-forms.

## Results

### Episomal Transfection of *P. Cynomolgi* using a DNA-construct Containing a Putative *P. cynomolgi* Centromere

**Figure 1:**
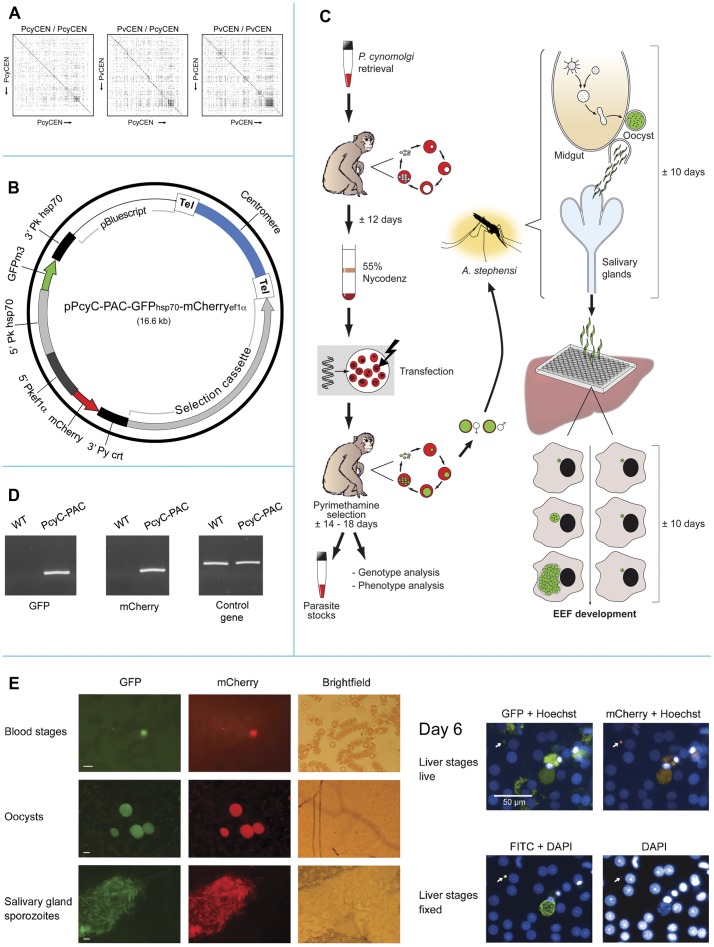
Construction and analysis of fluorescent *P. cynomolgi* using a novel centromere construct. (A) Dot matrix analysis of a *P. cynomolgi* and *P. vivax* putative centromere (PCEN). Graphical representation of a matrix analysis of a *P. cynomolgi* PCEN aligned against itself (*left*), *P. cynomolgi* PCEN against the *P. vivax* PCEN (*middle*) and *P. vivax* PCEN aligned against itself (*right*). The analysis was performed using Dotlet [46] as described before [22]. The diagonal line within each analysis represents sequence identity, and the diagonal line indicates repetitive regions within each PCEN. Note the absence of the diagonal in the repetitive regions of the *P. cynomolgi* and *P. vivax* alignment (Figure 1, *middle* panel). (B) Schematic representation of the pPcyC-PAC-GFP_hsp70_-mCherry_ef1α_ plasmid. The plasmid contains the *Tgdhfr-ts* selectable marker that confers resistance against pyrimethamine and two expression cassettes for constitutive expression of GFP and mCherry. Additionally, to maintain the plasmid throughout the life cycle, a putative *P. cynomolgi* centromere (PcyCEN) is included. (C) Schematic representation of the procedure used for transfection and analysis of *P. cynomolgi*. (D) PCR amplification of *gfp* and *mCherry* in PcyC-PAC-GFP_hsp70_-mCherry_ef1α_ (PcyC-PAC) blood stage parasites. Wild type gDNA of *P. cynomolgi* M served as negative control. For a control PCR primers for the *circumsporozoite protein (csp)* were used. For primers used, see Table S1. (E) GFP and mCherry expression throughout the life cycle of *P. cynomolgi.* GFP and mCherry expression in pPcyC-PAC-GFP_hsp70_-mCherry_ef1α_ transfected *P. cynomolgi* blood stage parasites (a ring and a trophozoite or gametocyte), in oocysts 5 days post mosquito feeding and in salivary gland sporozoites 12 days post feeding. In the Brightfield panel two salivary gland lobes can be distinguished; only one lobe contains sporozoites. In the panel on the right GFP and mCherry expression is shown in Hoechst 33342 stained day 6 liver stages. Note the autofluorescence of hepatocytes in the GFP channel in contrast to the mCherry channel. A small uninucleate (arrow) and a large multinucleate liver stage are visible, confirmed by staining of fixed parasites with anti-HSP70 antibodies (*lower right panel*). White bars correspond to 10 µm (blood and mosquito stages) and 50 µm (liver stages).

In the absence of robust and efficient methods to generate transgenic *P. cynomolgi* parasites by double crossover integration of DNA constructs into the genome we aimed for generation of transgenic parasites using episomal transfection. A disadvantage of episomal transfection is that transgenic *Plasmodium* parasites rapidly loose circular DNA-constructs during propagation in the absence of drug pressure due to uneven segregation of these constructs during mitosis [23], [24]. However when circular (and linear) DNA constructs contain *Plasmodium* centromeric sequences, they are stably segregated and maintained during propagation throughout the complete life cycle in the absence of drug selection pressure [22], [25]. With the aim to create stably fluorescent transgenic *P. cynomolgi* liver stages we therefore decided to include a centromeric sequence in our transfection construct. We first transfected *P. knowlesi*, a close relative of *P. cynomolgi* and the only non-human primate parasite that allows easy transfection and selection of genetically modified mutants *in vitro* [26], [27], with the centromere-containing *P. berghei* L-PAC construct [22]. When drug pressure was removed from the cultures, the L-PAC construct was rapidly lost. This indicates that the use of heterologous centromeric sequences does not result in stable maintenance of episomal constructs in *P. knowlesi,* similar to what has been reported in *P. berghei* [22]. Based on these observations we decided to first identify a putative *P. cynomolgi* centromere for inclusion in DNA constructs for subsequent *P. cynomolgi* transfection. Using primers based on a putative centromere from *P. vivax* (see Materials and Methods and Table S1), a 2.3 kb product was amplified from *P. cynomolgi* genomic DNA and sequenced (deposited at GenBank; accession number JQ809338). Sequence analysis showed a 93.8% A/T rich region with a core and a repetitive region as determined by Dotlet analysis (Figure 1A), characteristics which are indicative of a centromeric region [22]. A BLAST2 comparison of the sequence of the putative *P. vivax* centromeric region PvCEN (located between the genes PVX_113710 and PVX_113720) with the amplified, putative *P. cynomolgi* centromere, PcyCEN, showed an identity of 80% and Dotlet analyses revealed that the sequence identity was restricted to the core region, analogous to what has been described for rodent malaria centromeres [22] (Figure 1A).

In order to generate fluorescent *P. cynomolgi* liver stage parasites, we then generated construct pPcyC-PAC-GFP_hsp70_-mCherry_ef1α_ that includes the 2.3 kb centromeric region of *P. cynomolgi* (Figure 1B), a drug-selectable marker cassette [20] and two reporter genes *gfp* and *mCherry* under the control of ‘constitutive’ promoters. In the absence of genome data for *P. cynomolgi* we used the promoter regions of two *P. knowlesi* genes, *hsp70* (PKH_051230) and *ef1α* (PKH_111400). For both the *Plasmodium hsp70* and *ef1α* genes evidence has been found for constitutive expression throughout the life cycle [19], [28]. In addition we selected *hsp70* as we had found that anti-HSP70 antibodies stain *P. cynomolgi* liver stage cultures, including hypnozoite-forms.

The final construct, pPcyC-PAC-GFP_hsp70_-mCherry_ef1α_ (a circular artificial chromosome containing a homologous centromere and two marker genes) was used to transfect blood stages of *P. cynomolgi*. Because monkeys are necessary for these experiments, the procedure requires a robust system for collection and transfection of blood stages. Therefore we first optimised the enrichment procedure for *P. cynomolgi* blood stages (see Materials and Methods) resulting in a protocol (Figure 1C) that includes purification of infected blood cells by Nycodenz-density centrifugation [29], yielding a parasite preparation containing ≥95% red blood cells (rbc) infected with trophozoites and young schizonts. Subsequently 2×10^7^ Nycodenz-purified, infected rbc were transfected with the pPcyC-PAC-GFP_hsp70_-mCherry_ef1α_ construct using the Nucleofector technology [30] and these parasites were intravenously inoculated into a recipient monkey directly after transfection. Seven days post infection, the first parasites were observed and a day later pyrimethamine treatment was started to select transfected parasites. At day 13 a blood infection became patent again and at day 18, at a 2.4% parasitemia, the monkey was bled for preparing cryo-preserved parasite stocks and *ex vivo* mosquito feeding. In addition at day 20 blood was collected for a second mosquito feeding and for parasite genotyping and phenotyping. Genotype analysis by amongst others diagnostic PCR analysis (Figure 1D) showed the presence of the intact pPcyC-PAC-GFP_hsp70_-mCherry_ef1α_ constructs in the parasites. Moreover, fluorescence microscopy showed GFP- and mCherry-expression in blood stages of the PcyC-PAC-GFP_hsp70_-mCherry_ef1α_ parasites (Figure 1E). A global analysis of Hoechst-stained blood stages showed that most, if not all, trophozoites and schizonts were GFP- and mCherry positive. These results show that transgenic *P. cynomolgi* parasites can be selected that are episomally transfected using constructs containing a centromeric region. In addition it shows that the *hsp70* and *ef1α* 5′UTR regions of *P. knowlesi* can drive expression of reporter genes in *P. cynomolgi*. These PcyC-PAC-GFP_hsp70_-mCherry_ef1α_ parasites have been used for analysis of liver stage development (see below). The same procedure as for generation of PcyC-PAC-GFP_hsp70_-mCherry_ef1α_ parasites has now been applied in our laboratory in five independent experiments and in all experiments we successfully selected transgenic *P. cynomolgi* parasites (data not shown), indicating that this procedure as shown in Figure 1C is a robust system for *P. cynomolgi* transfection.

### pPcyC-PAC-GFP_hsp70_-mCherry_ef1α_ is stably Maintained in *P. cynomolgi* and the *P. knowlesi ef1α* and *hsp70* Promoters are Active Throughout the *P. cynomolgi* Life Cycle

To analyse maintenance of the DNA construct in parasites throughout the life cycle, we infected *Anopheles stephensi* mosquitoes with the PcyC-PAC-GFP_hsp70_-mCherry_ef1α_ parasites as described above. The mean number of oocysts 7 days after feeding in 10 independent experiments showed a wide variation ranging from 2 to >200 oocysts. This variation in oocyst production was also observed in *A. stephensi* that had been fed with wild type *P. cynomolgi*. Also sporozoite production of PcyC-PAC-GFP_hsp70_-mCherry_ef1α_ parasites was comparable to that of wild type parasites (a mean of 54,309 transgenic salivary gland sporozoites/mosquito derived from 10 transmission experiments compared to a mean of 53,125 wild type sporozoites/mosquito derived from 15 transmissions). Fluorescence microscopy of infected midguts and salivary glands revealed the presence of brightly fluorescent oocysts and salivary gland sporozoites, expressing both GFP and mCherry (Figure 1E). A limited survey indicated that, similar to centromere constructs in *P. berghei* [22], the majority of oocysts and sporozoites were fluorescent. In blood stages, oocysts and sporozoites, we never observed parasites that did not express mCherry and GFP simultaneously, indicating the constitutive nature of the promoters driving expression of the fluorescent markers. The normal oocyst and sporozoite production by PcyC-PAC-GFP_hsp70_-mCherry_ef1α_ indicates that expression of the reporter proteins does not grossly affect parasite development in the mosquito.

**Figure 2:**
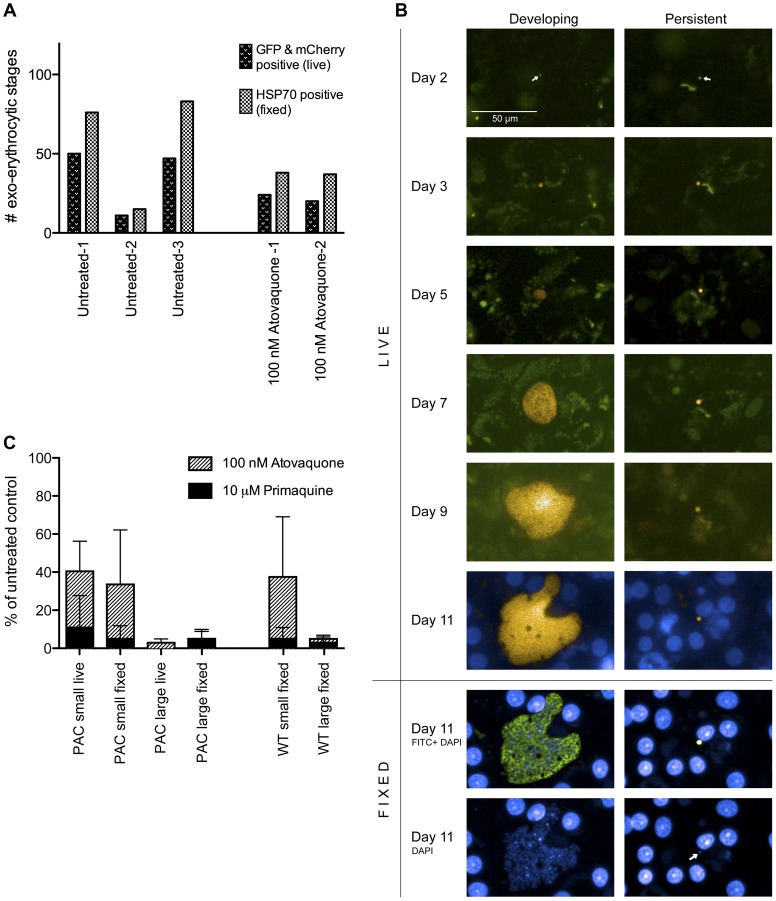
Identification and characterisation of *P. cynomolgi* hypnozoite-forms. (A) Exo-erythrocytic forms (EEF) counted live (GFP and mCherry positive) and fixed (after HSP70 staining), 6 days post hepatocyte infection. Total EEF numbers were counted in ‘untreated’ wells containing complete medium (three experiments, at least five fields counted per experiment) or wells that contained medium with 100 nM atovaquone throughout (two experiments, ten fields counted per experiment). (B) Real time monitoring of development of *P. cynomolgi* liver stages. Overlay of mCherry and GFP pictures taken at regular time intervals of *P. cynomolgi* liver stages. The left panel shows growth of a *P. cynomolgi* liver schizont towards its development into a multinucleate schizont. The right panel shows the presence of a small persistent liver stage parasite over a period of 11 days. Fixation and staining with anti-HSP70 antibodies and DAPI confirmed the presence of a multinucleate liver schizont and a small uninucleate parasite. The white bar corresponds to 50 µm. (C) Percentages of *P. cynomolgi* EEFs relative to EEF in untreated control wells post drug. *P. cynomolgi* wild type and PcyC-PAC-GFP_hsp70_-mCherry_ef1α_ (PcyC-PAC) liver stage cultures were harvested at day six post hepatocyte infection following continuous treatment with 100 nM atovaquone or 10 µM primaquine. Small uninucleate and developing (‘large’) parasites were counted on a high-content imaging system (PerkinElmer) according to custom made algorithms for live or fixed liver stage parasites using Harmony software (see Materials and Methods). Parasite counts were compared to EEF counts from untreated control wells and shown as percentages. Percentages of atovaquone and primaquine treated parasites are depicted superimposed. Live measurements of untreated PcyC-PAC control wells showed total numbers of EEF per well ranging from 16–377 (average number 132). For fixed measurements of PcyC-PAC, untreated control wells contained total EEF numbers ranging from 26–377 (average number 258) and for fixed WT measurements, control wells contained total EEF numbers ranging from 58–382 (average number 212). The results of three independent *P. cynomolgi* in vitro liver stage assays are represented as mean with SD.

We next analysed development of PcyC-PAC-GFP_hsp70_-mCherry_ef1α_ parasites during liver stage development using *in vitro* cultures of rhesus primary hepatocytes. Fluorescence microscopy analysis of live parasites at day 6 post infection with sporozoites shows maturing forms that are characterised by multiple nuclei (Hoechst staining) and their large size (Figure 1E). In addition, small uninucleate forms are present at day 6 (Figure 1E, arrow). Both the multinucleate and uninucleate forms are GFP and mCherry-positive and both forms stain with anti-HSP70 antibodies after fixation of the cells (Figure 1E). Because of the high background fluorescence of primary hepatocytes using standard FITC filter settings, as has also been reported previously [31], GFP expression in liver stages is sometimes hard to detect. However, careful analyses using long pass filters (see Materials and Methods) allowed the discrimination of GFP from background fluorescence and showed that mCherry-positive multinucleate and uninucleate forms were also GFP-positive. To determine whether all liver stage parasites expressed mCherry/GFP, using images taken by the Operetta High Content Imaging System (PerkinElmer), total numbers of mCherry/GFP expressing EEF were counted and compared to the total number of EEF from the same fields as assessed by HSP70 staining of fixed parasites. Figure 2A shows the results of the counts, derived from three independent experiments, counting ≥5 fields per experiment. Calculating the percentage of EEF that expressed mCherry/GFP revealed that a mean of 66% of EEF expressed mCherry/GPF (range 57–73%), indicating that the majority of parasites retain the construct during mosquito transmission and liver stage development, comparable to stable maintenance of centromere-containing constructs in mosquito transmission and hepatocyte infection of *P. berghei* [22]. A strong indicator for the presence of hypnozoites is their resistance to atovaquone [12]. To determine whether these stages were expressing mCherry/GFP, we treated liver stage cultures with 100 nM atovaquone to kill all developing parasites. Again, the total numbers of mCherry/GFP expressing EEF were counted and compared to the total number of EEF (only small forms in this case) from the same fields as assessed by HSP70 staining of fixed parasites. The total small form EEF counts from two independent experiments (counting 10 fields per experiment) are depicted in Figure 2A in the right panel. This showed that in the two experiments 54% and 63% of the atovaquone resistant EEF expressed GFP/mCherry, similar to the total number of untreated EEF that express GFP/mCherry. Thus we conclude that both small and developing EEF can consistently be detected by either antibody staining or live imaging of GFP/mCherry.

The Operetta system not only provides the opportunity to analyse fluorescent images taken at different time points but also allows for automated counting of parasites that are recognized based on different characteristics of the cells, i.e. their morphology and their fluorescence intensity. Automated counting HSP70-stained PcyC-PAC-GFP_hsp70_-mCherry_ef1α_ multinucleate and uninucleate parasites at day 6 post infection showed a mean percentage of infected liver cells of 0.34% (range 0.24% to 0.48%; 3 exp; 13 wells in total). This infection rate is comparable to the mean infection rate (0.39%) of wild type parasites that were present in the same plates (range 0.25% to 0.71%; 11 wells in total). These experiments also showed that for the transgenic parasites, the percentage of small stages ranged from 57–65% and for wild type parasites this was 57–63%. This averaged for both to a ratio of 39% multinucleate versus 61% uninucleate parasites, indicating that there was no difference in this ratio due to the presence of the centromeric construct. The percentage of small forms in *P. cynomolgi* liver stage cultures can vary from experiment to experiment *(Zeeman et al., in preparation)* and may, amongst others, depend on the quality of the batch of primary hepatocytes used.

These results indicate that i) expression of the reporter proteins does not affect invasion of liver cells and further development of the transgenic parasites, ii) stable maintenance of the centromeric construct enables the production of high percentages of fluorescent *P. cynomolgi* liver stage parasites, and iii) the heterologous promoters are active throughout the life cycle.

### Analysis of Developing *P. cynomolgi* Liver Stages and Hypnozoite-forms by Live Imaging

As described above we observed both multinucleate and small uninucleate parasites at day 6 after infection of the primary rhesus hepatocytes with PcyC-PAC-GFP_hsp70_-mCherry_ef1α_ sporozoites, indicating that a percentage of parasites arrest development. To analyse the development of these different forms we evaluated liver stages by live imaging throughout their development in culture. At day 2 only small parasite forms are present that are hardly visible/detectable by fluorescence microscopy. Between days 2 and 3 these small forms increase in size. After day 3 we distinguish two different forms in the cultures; forms that do not further increase in size and which remain uninucleate (Figure 2B, right column) and forms that increase in size and start nuclear division (at day 3 to 4), which progress to large multinucleate schizonts at day 11 (Figure 2B, left column). These large parasites appear with large vacuoles similar to what has been described for mature *P. yoelii* liver stage schizonts [13]. The persisting, small and uninucleate forms resemble the hypnozoite-forms described previously [12].

### The Hypnozoite-forms are Resistant to Atovaquone but are Killed by Primaquine

A characteristic of hypnozoites is their resistance to atovaquone treatment while they are killed by primaquine [12]. To further investigate the nature of the small fluorescent forms in our cultures, we treated cultures of wild type and PcyC-PAC-GFP_hsp70_-mCherry_ef1α_ with atovaquone and primaquine at concentrations that kill developing *P. cynomolgi* liver stage parasites [12]. Treatment was started three hours after adding the sporozoites to the primary rhesus hepatocytes and at day 6 after sporozoite infection, PcyC-PAC-GFP_hsp70_-mCherry_ef1α_ and wild type parasites were counted using the Operetta. Automated counting of fixed cells (based on their morphology and anti-HSP70 antibody staining) showed that, as expected, both PcyC-PAC-GFP_hsp70_-mCherry_ef1α_ and wild type developing liver stages were killed by the concentrations of atovaquone and primaquine used (Figure 2C). Small forms of both transgenic and wild type parasites were killed by primaquine, while a significant population of small forms was still present after atovaquone treatment (Figure 2C). These data show that there was no difference in drug activities against the transgenic liver stage parasites compared to the wild type parasites and suggest the presence of hypnozoite-forms in these cultures, analogous to what has been reported before [12].

For automated counting of live cells we developed a custom-adapted Harmony script based on mCherry and GFP fluorescence intensity and morphology (Perkin Elmer, see Materials and Methods). Counting of live PcyC-PAC-GFP_hsp70_-mCherry_ef1α_ cells showed comparable results to the counting of fixed parasites (Figure 2C). Treatment with primaquine resulted in the disappearance from the cultures of not only the large, developing stages but also of the small persistent forms. In contrast, these fluorescent small, persistent forms remained present in the cultures that were treated with atovaquone (Figure 2C). This was confirmed by a manual comparison of the live and fixed images obtained from atovaquone treated cultures (Figure 2A). The insensitivity to atovaquone and their killing by primaquine strongly supports the conclusion that these small, fluorescent parasites that persist for prolonged periods in culture, are hypnozoite-forms. In addition these results demonstrate that the transgenic parasites can be used in drug assays with live imaging read out.

### Hypnozoite-forms can be Isolated by Flow Sorting

**Figure 3:**
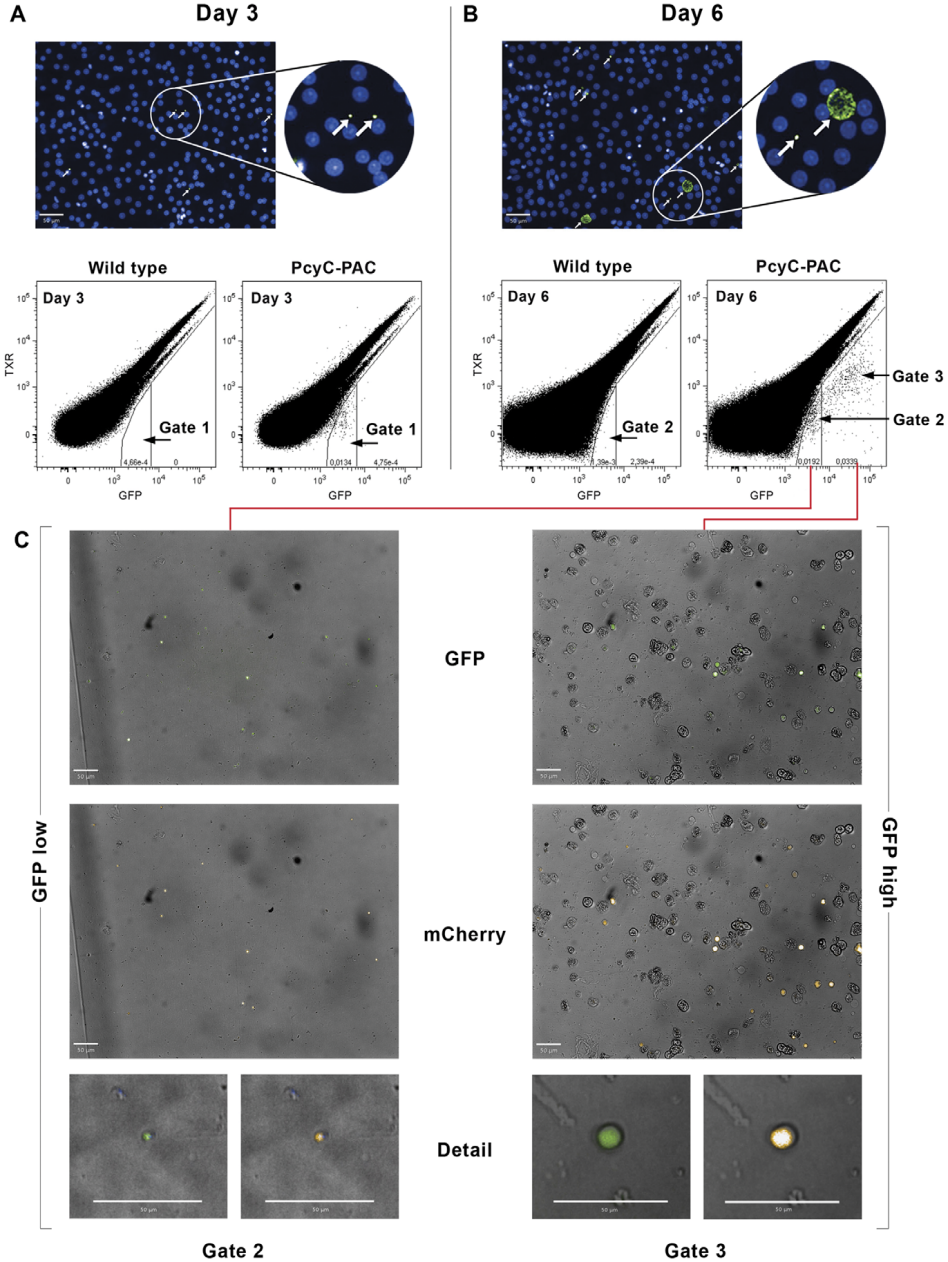
Flow cytometry and cell sorting of *P. cynomolgi* liver stage parasites, including hypnozoite-forms. (A) Liver stage parasites used for flowcytometry as detected by anti-HSP70 antibodies 3 days and (B) 6 days post hepatocyte infection. White bars correspond to 50 µm. Note that day 3 cultures contain uniform small parasites while day 6 cultures contain both small and large liver stages (arrows). Flow cytometric plots of PcyC-PAC-GFP_hsp70_-mCherry_ef1α_ (PcyC-PAC) *P. cynomolgi* liver stage parasites show a single GFP positive population compared to wild type parasites 3 days post hepatocyte infection (A, Gate 1) and two GFP positive populations 6 days post hepatocyte infection (B, Gates 2 and 3). The y-axis represents the PE-Texas Red Channel (for detection of autofluorescence), while the x-axis represents the GFP signal. (C) Post-sorting images of PcyC-PAC-GFP_hsp70_-mCherry_ef1α_ *P. cynomolgi* liver stage parasites ‘GFPlow’ (Gate 2) and ‘GFPhigh’ (Gate 3) parasites sorted at day 6 post hepatocyte infection. The upper panel shows a GFP/Brightfield overlay while the lower panel shows mCherry/Brightfield overlay. The panels below show close-ups of the sorted parasites revealing the size differences between the ‘GFPlow’ and ‘GFPhigh’ populations. White bars correspond to 50 µm.

The small size and low numbers of the hypnozoite-forms in culture, in addition to their intracellular location in a large host cell, hampers detailed analysis of the biology of these hypnozoite-forms, for example it excludes RNAseq or proteome analyses of these stages. We therefore investigated whether it was possible to purify these stages by flow sorting based on their fluorescence characteristics. We first analysed GFP-fluorescence intensity by flow cytometry of infected primary rhesus hepatocytes at day 3 and day 6 after infection of the cultures with PcyC-PAC-GFP_hsp70_-mCherry_ef1α_ sporozoites. At day 3 a clear population could be distinguished that showed an increased GFP-fluorescence intensity (cells in Gate 1 in Figure 3A) compared to uninfected hepatocytes. This population represents hepatocytes infected with GFP-positive parasites, as this population is absent in parallel cultures of wild type liver stages (Figure 3A). At day 6 infected cells were observed with the same GFP-fluorescence intensity as on day 3 (cells in Gate 2 in Figure 3B) and cells with increased GFP intensity (cells in Gate 3 in Figure 3B). The percentage of infected cells with low GFP intensity (Gate 2) of the total infected cells (Gate 2 and 3) was 57% (range 47% to 63%; 3 exp.) and is in line with data from manual (fluorescence microscopy) and automated counting (Operetta System) of hypnozoite- and developing-forms, as also reported above. To further investigate the two different populations, cells were flow sorted from Gate 2 and Gate 3 as shown in Figure 3B and these cells were imaged using the Operetta system. Cells sorted from Gate 2 (three independent experiments) were GFP and mCherry-positive parasites of which on average 97% (range 90% to 100%; 3 exp.) were small uninucleate parasite stages (hypnozoite-forms), whereas cells from Gate 3 were mainly developing liver stage parasites (35%–83%; 3 exp.; Figure 3C). These results demonstrate that it is possible to purify a population of small, uninucleate parasites that includes hypnozoite-forms. Most sorted parasites, both the small forms and developing forms, were extracellular. This may be due to the small nozzle size used during the sorting procedure, and/or to increased hepatocyte fragility resulting from collagenase treatment. Preliminary data suggest that trypsinization of the cells instead of collagenase treatment appears to improve the integrity of the cells and overcome the problems associated with host cell rupture.

Following this method, we estimate to obtain some 5,000 sort events for small as well as developing forms from an optimal large-scale experiment. Combining purified material from several experiments, each requiring the infection of a rhesus monkey with the transgenic parasite, should yield sufficient material for subsequent –omic analyses, similar to described by *Tarun et al.*, who used some 40,000 purified rodent malaria EEF for such analyses [36].

## Discussion

At present extremely little is known about hypnozoites, the dormant malaria liver stages, mainly due to the technical hurdles associated with working with *P. vivax* and the lack of tools to study the biology of this cryptic parasite stage [2], [8]. Here, we report the development of novel tools to dissect hypnozoite biology using *P. cynomolgi* as a hypnozoite-forming model malaria parasite. An advantage of working with this species is the readily and reproducible access to sporozoites, which is required for studies on liver stages using *in vitro* cultivation technologies. Episomal transfection of *P. cynomolgi* has already been reported for *P. cynomolgi* [20], but we have now developed a novel DNA-construct containing a centromeric region in order to maintain episomes throughout the life cycle and to express fluorescent-proteins in the liver stages. This is the first time that a centromere-containing construct is used in a non-rodent malaria parasite to express (reporter) proteins in liver stages. Not all liver stage parasites could be visualized, indicating that in the absence of drug selection in the mosquito, some of the constructs may have been lost. This could be overcome by integrating the marker genes into the genome using gene insertion. Although published by others [21], this technology has not yet been fully established in our laboratories. Nonetheless, the presence of the centromere enabled expression of marker genes in a significant proportion of the liver stage parasites, including hypnozoite-forms. This shows that episomally maintained centromere-containing constructs provide a new, simple approach for (over-) expression of proteins in many developmental stages, including the liver stages. Moreover, next to stable maintenance, in the rodent parasite *P. berghei* [22] and the human parasite *P. falciparum* [25] it has been shown that transfection with centromere-containing constructs (linear or circular) is highly efficient. Given the current limitations for in vitro cultivation of *P. vivax* blood stages and transfection of *P. vivax* [32], it might be worthwhile to explore whether this approach can be applied for this species as well.

Development of the PcyC-PAC-GFP_hsp70_-mCherry_ef1α_ parasites appeared to progress normally throughout the *in* *vivo* life cycle, both in primates and mosquitoes, indicating no significant loss of fitness of the transgenic parasites due to expression of the two reporter-proteins and/or the presence of the centromere-containing construct. Previously it has been shown that *P. berghei* parasite development in the mosquito can be affected when two reporters, GFP and mCherry, were expressed under the control of the same constitutive promoter, *ef1α* [15]. The majority of these transgenic parasites arrested during the oocyst stage. We have not observed these problems with our construct that contains the 5′UTRs of *P. knowlesi hsp70* and *ef1α* to drive expression of GFP and mCherry, respectively, indicating that a combination of these two constitutive promoters for driving transgene expression is feasible throughout the life cycle.

*Hsp70*-driven expression of GFP was readily observed throughout the life cycle. Interestingly also salivary gland sporozoites were clearly GFP-positive. Although HSP70.1 has been detected in sporozoites before [28], its abundance is very low and also IFA analysis of *P. cynomolgi* sporozoites using anti-HSP70 antibodies provided evidence for low expression compared to liver stages. GFP expression in the PcyC-PAC-GFP_hsp70_-mCherry_ef1α_ sporozoites is in line with observations on transgenic *P. berghei* parasites that also showed *hsp70* promoter-driven GFP expression in sporozoites [33]. It is possible that the GFP seen in sporozoites is not a result of *hsp70* promoter activity in sporozoites but of carry-over of GFP from oocysts or midgut sporozoites, since GFP has been reported to have a relatively long half-life [34]. Alternatively, regulatory signals of the endogenous *hsp70* gene that control its expression in sporozoites may be absent in the transfection construct used leading to ‘aberrant’ expression of GFP in sporozoites.

The use of PcyC-PAC-GFP_hsp70_-mCherry_ef1α_ parasites provided the opportunity for a detailed analysis of *P. cynomolgi* development in hepatocytes. Hypnozoites of *P. vivax* type parasites are described as small uninucleate, persisting liver stage parasites that are not killed by most antimalarials but are sensitive to primaquine (and other 8-aminoquinolines) [35]. In hepatocyte cultures containing PcyC-PAC-GFP_hsp70_-mCherry_ef1α_ parasites we show the presence of small fluorescent parasite forms that were uninucleate and persistent, characteristics reminiscent of hypnozoites. Primaquine treatment resulted in the disappearance of these forms whereas in cultures treated with atovaquone, a drug that kills all pre-erythrocytic stages except hypnozoites [12], a significant population of small parasites remained alive. This indicates that these forms are indeed atovaquone-resistant hypnozoite-forms. Rapid deterioration of the primary hepatocyte cultures beyond two weeks prohibit witnessing reactivation of these hypnozoite-forms, if that would be possible at all in vitro, in the absence of possibly critical physiological factors from the host. Thus, in the absence of further markers for hypnozoites other than being small, persistent, uninucleate EEF resistant to atovaquone and capable of reactivation (the last of which we have not demonstrated), we prefer the term ‘hypnozoite-form’, to indicate that these in vitro cultured parasites contain most known characteristics of hypnozoites. The small number of small forms that were killed by atovaquone may represent a population of parasites that abort development for other reasons, similar to non-dividing pre-erythrocytic stages that are observed in cultures of *P. falciparum* liver stages [12].

The robust *P. cynomolgi* transmission platform and *in vitro* liver stage culture capability [12] (A.M. Zeeman, *in preparation*) provide important new tools for *in vitro* drug screening and bring studies aiming to investigate hypnozoite biology within reach. However, hepatocyte infection grades are maximally a few percent meaning that hypnozoite transcriptomics and proteomics will suffer from serious amounts of hepatocyte contamination. For the rodent malaria *P. yoelii*, FACS purification and subsequent transcriptome and proteome survey of liver stages using GFP expressing parasites has been described [13], [36]. Importantly here we demonstrate that a similar approach for *P. cynomolgi* is feasible. Beyond what has been achieved with *P. yoelii* that only produces developing liver stages, we have shown that we can isolate both developing liver stages and critically also hypnozoite-forms from infected hepatocyte cultures to great levels of purity and after scaling up this procedure, a detailed molecular characterisation of these stages should become feasible. In the absence of markers for hypnozoites, we cannot exclude that, next to hypnozoite-forms, a small parasite population that has aborted its development is purified alongside. Therefore, in future large-scale FACS purification experiments, one solution to avoid this may be to exploit an atovaquone treatment to kill such stages, as previously described [12].

Given the lack of knowledge of hypnozoites these novel tools for stable transfection of *P. cynomolgi*, visualisation of live liver stages and purification of hypnozoite-forms will most likely provide a wealth of information on these so far elusive parasite forms, including identification of novel targets for chemotherapy and for vaccine development.

## Materials and Methods

### Ethics Statement

All rhesus macaques (*Macaca mulatta*) used in this study were captive bred for research purposes and were socially housed at the BPRC facilities under compliance with the Dutch law on animal experiments, European directive 86/609/EEC and with the ‘Standard for humane care and use of Laboratory Animals by Foreign institutions’ identification number A5539-01, provided by the Department of Health and Human Services of the USA National Institutes of Health (NIH). Nonhuman primates were used because no other models (in vitro or in vivo) were suitable for the aims of this project. Besides their standard feeding regime, animals followed an environmental enrichment program in which, next to permanent and rotating non-food enrichment, daily an item of food-enrichment was offered to the macaques. All animals were daily monitored for health and discomfort. The local independent ethical committee constituted conform Dutch law (BPRC Dier Experimenten Commissie, DEC), approved all research protocols prior to the start and all experiments were performed according to Dutch and European laws. The Council of the Association for Assessment and Accreditation of Laboratory Animal Care (AAALAC International) has awarded BPRC full accreditation. Thus, BPRC is fully compliant with the international demands on animal studies and welfare as set forth by the European Convention for the Protection of Vertebrate Animals used for Experimental and other Scientific Purposes, Council of Europe (ETS 123), Dutch implementing legislation and the Guide for Care and Use of Laboratory Animals. The liver lobes were collected from monkeys that were euthanized in the course of unrelated studies (ethically approved by the BPRC DEC) or euthanized for medical reasons, as assessed by a veterinarian. Therefore, none of the animals from which liver lobes were derived were specifically used for this work, fully in accordance with the 3R’s, reducing the numbers of animals used. Euthanasia was performed under ketamine sedation (10 mg/kg) and was induced by intracardiac injection of euthasol 20%, containing pentobarbital. All intravenous injections and large blood collections were performed under ketamine sedation, and all efforts were made to minimize suffering.

### DNA Constructs

Based on synteny [37], primers were designed against *P. vivax* sequence containing a predicted centromere (sequence between PVX_113710 and PVX_113720). Upon PCR amplification of *P. cynomolgi* M strain genomic DNA with a lowered extension temperature as described earlier [38] using primers 2005 and 2006 (see Table S1 for a list of primers), a fragment of 2.3 kb containing a putative *P. cynomolgi* centromere was amplified. The fragment was cloned into pCR®-Blunt II-TOPO (Invitrogen) and sequenced. The sequence is deposited to Genbank under accession number JQ809338. Through a series of cloning steps 0.7 kb of the 3′UTR of *P. yoelli chloroquine resistance transporter (crt)* [39] (kindly provided by Dr. D. Fidock, *GFPmutant3* [40] and 0.7 kb of the 3′ UTR of *P. knowlesi heat shock protein 70 (hsp70)* were cloned into plasmid pD.D_Tm_.D. [41]. Using the Gateway® Vector Conversion System (Invitrogen) the plasmid was converted into a destination vector. Subsequently, *P. berghei* telomeric regions derived from plasmid C-PAC [22] were introduced. 1.5 kb of 5′UTR of *P. knowlesi hsp70* and 1.1 kb of 5′UTR of *P. knowlesi elongation factor1α* (*ef1α*) fused to *mCherry* were PCR amplified and introduced into plasmid pDONR221 P1-P5r and pDONR221 P5-P2 using the MultiSite Gateway Pro Plus system (Invitrogen) to generate entry clones pENTR 5′Pkhsp70L1-L5r and pENTR PkEF-mCherry L5-L2. These fragments were then simultaneously shuttled into the destination vector. Finally, the putative *P. cynomolgi* centromere was cloned in between the telomeric regions to generate plasmid pPcyC-PAC-GFP_hsp70_-mCherry_ef1α_ (Figure 1B). Primers used for amplifying the various parts of the construct are depicted in table S1. PCR products were sequenced for confirmation.

### Parasite Manipulations

To optimise the enrichment procedure for *P. cynomolgi* blood stage parasites, we collected *P. cynomolgi* infected blood at the BPRC from monkeys from unrelated experiments that had been ethically approved by the BPRC DEC, and tested it on 52, 55, 58 and 60% Nycodenz cushions (similar to what has been done in P. yoelii [42]). In three independent experiments this consistently yielded the highest purity levels of trophozoite/young schizont preparations at 55% Nycodenz and therefore this was chosen for enrichment of parasites for transfection. A *P. cynomolgi* M strain infection was initiated in a rhesus monkey (*Macaca mulatta*) by intravenous injection of 1×10^6^ blood stage parasites from a cryopreserved stock. At peak parasitemia (as monitored by reading Giemsa-stained thin blood films prepared from finger prick derived blood) heparin blood was taken and parasites, mainly young trophozoites, were purified on a 55% Nycodenz (Axis-Shield) cushion in PBS. After centrifugation at 300 g for 25 min at Room Temperature (low brake), a layer containing trophozoites at a purity of >95% was isolated and washed in RPMI 1640. Parasites were cultured overnight in complete medium (RPMI1640 containing 20% heat inactivated Human A+ serum and 15 µg/ml gentamicin) for further development and washed once in RPMI 1640. Subsequently 2×10^7^ *P. cynomolgi* parasites were resuspended in Human T-cell buffer, mixed with 10 µg of pPcyC-PAC-GFP_hsp70_-mCherry_ef1α_ and transfected using the Nucleofector device (Lonza, program U33). Immediately after the pulse, PBS was added to a total volume of 0.5 ml that was injected intravenously into a recipient monkey. One week later the monkey was positive for blood stage parasites and pyrimethamine treatment was initiated (1 mg/kg, orally on a biscuit every other day) to select for transfected parasites. Six days later a resistant parasite population emerged and at peak parasitemia blood was obtained for stocks, mosquito feeding and analyses. Mosquito feedings were performed on blood obtained from the recipient monkey from the transfection or, for further transmission experiments, donor monkeys were infected with thawed stocks of PcyC-PAC-GFP_hsp70_-mCherry_ef1α_ blood stage parasites and from day 2 onwards treated every other day with pyrimethamine until day 8. Patency usually occurs at day 7 and at parasitemias ranging from 0.2% to 1.7% (generally between days 11 and 14) mosquitoes were allowed to feed on two different days on blood obtained from the monkey.

### Mosquito Stages of *P. cynomolgi*

Two to five days old female *Anopheles stephensi* mosquitoes Sind-Kasur strain Nijmegen (Nijmegen UMC St. Radboud, Department of Medical Microbiology [43]) were fed on blood obtained from a monkey that had been infected with wild type or PcyC-PAC-GFP_hsp70_-mCherry_ef1α_ parasites using a glass feeder system. Mosquitoes were housed in climate chambers at 25**°**C and 80% humidity and fed regularly via cotton soaked in 5% D-glucose solution. Approximately one week after infection oocysts were counted and mosquitoes were given an uninfected blood meal to promote sporozoite invasion of the salivary glands. Salivary gland sporozoites were present from day 12 post feeding onwards. PcyC-PAC-GFP_hsp70_-mCherry_ef1α_ blood stages, oocysts and salivary gland sporozoites were viewed under a Nikon Microphot FXA fluorescence microscope using filters 485DF22/505DRLP/515EFLP for GFP expression and 560DF40/595DRLP/600EFLP for viewing mCherry and pictures were taken using a Nikon DS-5M digital camera.

### Primary Hepatocytes

Rhesus primary hepatocytes were isolated using a two-step enzymatic perfusion essentially as described [44] and resuspended in William’s B medium: William’s E with glutamax containing 10% fetal calf serum (FCS), 1% NEAA, 2% penicillin/streptomycin, 1% insulin/transferrin/selenium, 1% NaPyruvate, 50 µM β-mercapto-ethanol, and 10^−7 ^M dexamethasone (Alfasan, Woerden, The Netherlands). Hepatocytes were seeded into collagen coated (5 µg/cm^2^ rat tail collagen I, Sigma) 96-well Greiner cellstar plates at a concentration of 9×10^4^ cells/well or into 6-well Costar plates at a concentration of 2.25×10^6^ cells/well. Following attachment, the medium was replaced by William’s B containing 1% dimethylsulfoxide (DMSO) to prevent hepatocyte dedifferentiation.

### Sporozoite Inoculation

Between 14 and 28 days post mosquito feeding on *P. cynomolgi* infected blood salivary gland sporozoites were isolated and used for hepatocyte inoculation [45] at a concentration of 5×10^4^ sporozoites per well in 96-well plates or at 1.5–2×10^6^ sporozoites per well in 6-well plates. Immediately after sporozoite inoculation 96-well plates were spun at RT at 500×g for 10 min and 6-well plates were left at room temperature for 2 h to settle the sporozoites. After placing the plates for two to three hours in a 37**°**C incubator at 5% CO_2_ to allow for sporozoite invasion, medium was refreshed. From then onwards, medium was refreshed every other day until the cultures were analysed. For drug treatment, atovaquone or primaquine were added to the *P. cynomolgi* liver stage cultures at the time of the first medium exchange and added each time medium was refreshed until fixation in cold methanol at day 6 post sporozoite inoculation.

### Visualisation of Exo-erythrocytic Forms (EEF)

Methanol-fixed EEF were stained with antibodies directed against *P. cynomolgi* HSP70.1 (A.M. Zeeman, in preparation) as described [12]. To view nuclei of live parasites, Hoechst 33342 (Invitrogen) was added to the cultures at 10 µg/ml and cultures were viewed with the Operetta or with the Leica DMI6000 inverted microscope using a Leica I3filter (Excitation filter BP450–490/Dichromatic Mirror 565/Suppression filter BP600/40) to visualise GFP and a N3 filter (Excitation filter BP546/12/Dichromatic Mirror 510/Suppression filter LP 515) to visualise mCherry in EEF. EEF numbers were determined with a high-throughput high-content imaging system (Operetta, Perkin-Elmer).

### Custom Scripts for Live and Fixed *P. cynomolgi* EEF

Using the Harmony software custom scripts for detection of *P. cynomolgi* live and fixed EEF were developed empirically. For fixed parasites, a comparison of more than 100 wells from different plates showed that numbers and the proportion of small versus multinucleate forms were similar for manual and automated counting. For live parasites, comparisons were made between automated Operetta counts, manually counted EEF and fixed counted EEF. When necessary, scripts were adjusted to obtain the best match. For detection of live *P. cynomolgi* EEF the following criteria were used: threshold for the mCherry image region was set at 0.80. Fluorescent populations were counted when the ratio of mCherry versus autofluorescence was higher than 1.2 in combination with a mean Hoechst intensity >60 and a mean GFP intensity >250. Small EEF were characterised by a fluorescent image region of >3 µm^2^ and <20 µm^2^. Large EEF had a fluorescent image region of ≥20 µm^2^. For detection of fixed *P. cynomolgi* EEF, where staining with antibodies against HSP70 and subsequent secondary labelling with anti-FITC antibodies had been performed the criteria for *P. cynomolgi* EEF were as follows: the threshold for the Fluorescein image region was 0.83. Fluorescent populations were counted at a ratio FITC-Autofluorescence >5, a DAPI intensity >50 and a Fluorescein intensity image region >1000. Small EEF were characterised by a fluorescent image region of >12 µm^2^ and <30 µm^2^. Large EEF had a fluorescent image region of ≥30 µm^2^.

### Flow Cytometric Analyses and EEF Sorting

For FACS, hepatocyte cultures infected with wild type or pPcyC-PAC-GFP_hsp70_-mCherry_ef1α_ sporozoites were harvested by Collagenase treatment (Collagenase IV, Sigma, 5 min. at 37**°**C). Cells were washed twice in William’s B medium and analysed and sorted using a BD FACSAria flowcytometer equipped with a 488 nm Coherent® Sapphire™ solid state 20 mW Laser. Data analyses were performed using FlowJo Version 9.4.10 (TreeStar, Inc., Ashland OR, USA). The machine was equipped with a 100 µM nozzle for sorting.

## Supporting Information

Table S1 **Sequences of oligonucleotides used for construction and analysis of pPcyC-PAC-GFP_hsp70_-mCherry_ef1α._**
(DOC)
