# Supplementary material for: Transgenic Fluorescent Plasmodium cynomolgi Liver Stages Enable Live Imaging and Purification of Malaria Hypnozoite-Forms
Source: PLoS One. 2013 Jan 24;8(1):e54888. doi: 10.1371/journal.pone.0054888 (PMC3554669; doi:10.1371/journal.pone.0054888)
Supplement: Table S1 — Sequences of oligonucleotides used for construction and analysis of pPcyC-PAC-GFPhsp70-mCherryef1α. (DOC) [file pone.0054888.s001.doc]

| **Gene** | **Oligonucleotide number** | **Sequence 5’ – 3’** | **Remarks** |
| --- | --- | --- | --- |
| GFP mutant 3 | 1920 (F) | cgAGATCTATGTCTAAAGGAGAAGAACTTTTC | Contains a *Bgl*II site (underlined) |
|  | 1921 (R) | cggGGATCCTTATTTGTATAGTTCATCC | Contains a *Bam*HI site (underlined) |
| *P. knowlesi hsp70* (PKH_051230) 3’ UTR | 1922 (F) | cggGGATCCacggagtcaaatccaaagcatc | Contains a *Bam*HI site (underlined) |
|  | 1923 (R) | cggGGATCCcaatgtaacaataatttttatcac | Contains a *Bam*HI site (underlined) |
| *P. knowlesi hsp70* (PKH_051230) 5’ UTR | 1932 (F) | *ggggacaacttttgtatacaaagttgt*ATTACCCCTTATACATAGGCAATAAG | Contains an attB5r site (italics) |
|  | 1933 (R) | *ggggacaagtttgtacaaaaaagcaggctTT*GAATTTTCAAGAGGGTGTGAAAAAG | Contains an attB1 site (italics) |
| *P. knowlesi ef1alpha* (PKH_111400) 5’ UTR | 2053 (F) | *ggggacaactttgtatacaaaagttgGG*CCACATATGAAAAGTGC | Contains an attB5 site (italics) |
| mCherry | 1936 (F) | *ggggacaactttgtataataaagttg*ATGGTGAGCAAGGGCGAGGAG | Contains an attB3 site (italics) |
|  | 1937 (R) | *ggggaccactttgtacaagaaagctgggta*TTACTTGTACAGCTCGTCCATG | Contains an attB2 site (italics) |
| *P. cynomolgi csp* 3’ UTR | 2031 (F) | ccGATATCGTAGCTGGCATCCATTTTTTC | Contains a *Eco*RV site (underlined) |
|  | 2032 (R) | ccATCGATTAGCAACTTCTCAACTAAGGC | Contains a *Cla*I site (underlined) |
| PcyCEN | 2005 (F) | CAAATAATCTAAAGAGAAATGC |  |
|  | 2006 (R) | AACAACAGCAAAATCAGTCTAC |  |
